# Does Self-Assessed Health Reflect the True Health State?

**DOI:** 10.3390/ijerph182111153

**Published:** 2021-10-23

**Authors:** Pavitra Paul, Ulrich Nguemdjo, Natalia Kovtun, Bruno Ventelou

**Affiliations:** 1 Centre de Sciences Humaines (CSH, UMIFRE n°20), a Network of Research Units of the National Centre for Scientific Research (CNRS) and the French Ministry of European and Foreign Affairs, New Delhi 110011, India; 2 Department of Public Health, Yerevan State Medical University, Yerevan 0025, Armenia; 3 CNRS, AMSE, Aix-Marseille Université, 13001 Marseille, France; ulrich-boris.nguemdjo-kamguem@univ-amu.fr (U.N.); bruno.ventelou@univ-amu.fr (B.V.); 4 Laboratoire Population Environnement Développement, Aix-Marseille Université, 13001 Marseille, France; 5 Department of Statistics and Demography, Taras Shevchenko National University of Kyiv, 03022 Kiev, Ukraine; kovtun_natali@ukr.net

**Keywords:** clinical morbidity, endogeneity, perceived health, recursive, semi-ordered, Russia

## Abstract

Self-assessed health (SAH) is a widely used tool to estimate population health. However, the debate continues as to what exactly this ubiquitous measure of social science research means for policy conclusions. This study is aimed at understanding the tenability of the construct of SAH by simultaneously modelling SAH and clinical morbidity. Using data from 17 waves (2001–2017) of the Russian Longitudinal Monitoring Survey, which captures repeated response for SAH and frequently updates information on clinical morbidity, we operationalise a recursive semi-ordered probit model. Our approach allows for the estimation of the distributional effect of clinical morbidity on perceived health. This study establishes the superiority of inferences from the recursive model. We illustrated the model use for examining the endogeneity problem of perceived health for SAH, contributing to population health research and public policy development, in particular, towards the organisation of health systems.

## 1. Introduction

The self-assessed health (SAH) measure results from an “ordered” response of the individual on a qualitative (subjective) perception of her/his own health status (perceived health), although the subjective nature of SAH remains questionable in the literature [1,2,3,4,5]. If all individuals have a similar perception of health status, SAH would reflect ‘true’ health and could therefore serve as a valid indicator. However, problems occur when individuals vary in their reporting behaviour and, as a consequence, SAH deviates from the underlying ‘true’ health. For instance, the same clinical health condition is acknowledged differently according to individual characteristics and is determined by the cultural and historical context, social position, and health experiences of the individual [1,6]. A potential explanation for such varying assessments lies in different people’s varying ability to adapt to ill health [2,7,8]. People who have been ill for a long period of time may report levels of health that are much better than those of individuals suffering from the same illness for the first time, which then leads to the differential reporting of SAH among those with an objective health status. We attempt to examine this further.

An abundance of literature seeks to explain the variations in SAH [9,10]. Confronted by a given measurement of ‘true’ health, SAH seems to be associated with sociodemographic, socioeconomic, behavioural, psychosocial, and chronic health conditions [11,12,13,14,15,16]. Tay et al. [17] established a relationship between SAH and sociodemographic factors, wherein gender and material circumstances consistently and independently predict SAH. Hernàndez-Quevedo et al. [18] found that reporting heterogeneity is associated with socioeconomic strata; this differs from the concept of social class, which refers to social groups arising from interdependent economic relationships among people [19]. Au and Johnston [9] found associations between health dimensions (including history of illness) and SAH for an average level of perceived health. Lindeboom and van Doorslaer [20] found evidence of age and gender-related reporting bias in SAH. Krause and Jay [21] found that people of different age groups tend to think of health differently when making evaluations: older respondents were more likely to use specific health problems (hypertension) as a reference point for their health, while younger people focused on their own physical functioning such as mobility issues and acute conditions. Lastly, and probably the most troubling result, some chronic medical morbidity was found to be positively associated with SAH for late middle-aged and elderly non-institutionalised people living in the northern part of the Netherlands [22]. A qualitative study [23] concluded that subgroup differences in SAH could be attributed to experiences with ill health.

Systematic differential health status reporting by subgroups of the population presents a serious problem to the validity of subgroup comparisons of health levels and thus to measure degrees of inequality. In particular, the possible sensitivity of SAH to the information previously delivered to the respondents by the health system is an acknowledged concern for the use of SAH metrics in health inequality measurements; this implies that the response of the individual is not independent from the individuals’ (or groups’) healthcare services utilisation [24], although such independency is precisely expected when SAH is used for evidence-informed policy development. Experience of interaction with the health system is not independent of socioeconomic strata and thus the thresholds of reported health status is often influenced by the frequency and quality of encounters for each specific individual with the respective health system [25,26].

Distinguishing the underlying cause of differences in reporting behaviour becomes a necessity to unveil the bias in the measurement of socioeconomic inequality in health. Although several studies have investigated factors that are likely associated with SAH and socioeconomic biases, we focus on finding the answer to the question of effect regarding clinical morbidity (diagnosed chronic disease) on different levels of the perceived health status. We also aim at examining the effect of duration of suffering from chronic disease on the perceived health status. However, considering that having clinical morbidity as a predictor of perceived health may raise an endogeneity problem when using a naive regression model, we introduce the application of the recursive semi-ordered probit model to control for endogeneity, as well as to examine the “causal” effect of the clinical morbidity on the perceived health in the direction of such relationship. The strength of this study concerns understanding the tenability of the construct of SAH by simultaneously modelling SAH and clinical morbidity, while operationalising a simultaneous binary and ordered probit-semi ordered approach [27] in healthcare and health policy research. Furthermore, our approach also contributes to the estimation of the distributional effect of clinical morbidity on different levels of perceived health. We describe the data and analysis strategy in Section 2. Section 3 presents the descriptive statistics and results from the application of the econometric models. We discuss the empirical evidence that emerged in Section 4 and conclude with the contributions of this study in Section 5.

## 2. Materials and Methods

Our data derives from 17 waves (2001–2017) of the Russian Longitudinal Monitoring Survey (RLMS) that captures repeated response on SAH and updates information on diagnosed chronic illness (considered as clinical morbidity in our study). SAH is the subjective response elicited from the structured question “How would you evaluate your health?” in an ordered scale (1 = very good (excellent), 2 = good, 3 = average, 4 = bad, and 5 = very bad), while diagnosed chronic illness indicates the diagnosis established by a physician. The multivariate distribution of the sample by gender, age, and urban/rural location compared quite well with the corresponding multivariate distribution of the Soviet/Russian census: there is usually a difference of only one percentage point or less between the two distributions—http://www.cpc.unc.edu/rlms (accessed on 21 February 2021). The RLMS (http://www.cpc.unc.edu/rlms, accessed on 21 February 2021) is an ongoing longitudinal household survey of the Russian Federation and the survey captures both the perceived health status and specific (objective) health status at the individual level (Table 1). The RLMS applies a multi-stage sampling method with precomputed cross-sectional post-stratification weights. These weights adjust not only for design factors but also for deviations from the census characteristics. Due to the decline in response rate in large cities, sample repair was done in Round 15 (2006; http://www.cpc.unc.edu/projects/rlms-hse/data, accessed on 21 February 2021).

Our data is a mix of cross-sectional and panel data (in which a segment of the households were followed over time, although only 313 individuals were present in all 17 waves), although the panel is not a balanced one. After omitting respondents with missing information on the outcome variables or covariates, the estimation sample included 145,239 observations of respondents aged 18 years and above from 79,795 households (Table 2).

Our analysis included adult individuals with clinical morbidity, i.e., heart disease, gastrointestinal disease, spinal disease, and other chronic disease. Furthermore, we also included individuals with assigned disabilities. A large number of missing information on the year of diagnosis for other diseases did not allow us to include other morbid conditions captured in the survey. Therefore, our variable of clinical morbidity was any one (and/or in combination) of the four specified diseases and the duration (the maximum duration of suffering with any one (and/or in combination) of these four diseases and the maximum duration of an assigned disability; Table 1). Table 1 also indicates the presence of clinical morbidity at different levels of perceived health. 

To control for response heterogeneity, we included in our analysis a vector of time-varying characteristics, i.e., age, educational attainment, marital status, and working status. We additionally controlled individual-level fixed effects, which represent time-invariant characteristics, i.e., settlement of residence (village, town, and city). We also included the response for income and overall life satisfaction in our study.

Our variable of interest (perceived health status) is an ordered categorical variable and the simple ordered probit model (naive model; details in Appendix A) could appear as a natural choice for assessing the effect of clinical morbidity on perceived health status. However, clinical morbidity, when considered as a determinant of perceived health status, raises an endogeneity issue concerning the affliction with clinical morbidity and perceived health status that is likely to be dependent and perhaps often influenced by the same unobservable confounders.

The standard instrumental variable (IV) approach is likely to produce inconsistent estimates when endogeneity stems from discrete regressors [27]. The classical ordered probit model assumes that errors in the latent regression equations for the selection mechanism and outcome variable follow a bivariate Gaussian distribution. The maximum likelihood (ML) estimator of an ordered probit model is known to be inconsistent if the unobservable factors affecting the outcome of interest are correlated with the unobservable factors affecting the selection mechanism. So, we applied a recursive semi-ordered probit model [27] under the assumption of normally distributed unobserved errors; we attributed a distribution to *β* with a mean, *b*_0_ = *E*(*β*) and a dispersion matrix, *σ*_2_*P*_0_ = *D*(*β*); this is, in effect, a Bayesian prior. The model is a recursive, simultaneous equation model. Using the sample of 145,239 adults, we simultaneously modelled perceived health and clinical morbidity. The model is recursive because $y_{1i}$, the observed binary realisation of the latent variable $y_{2i}^{*}$, appears on the right-hand side of Equation (2).

(1) $$y_{1i}^{*}=\sum_{j=1}^{7}x_{1ij}^{\prime }\beta_{1j}+\varepsilon_{1i}$$

where $y_{1i}$ is a vector of clinical morbidity (1 = yes; 0 = no) and $x_{1ij}^{\prime }$ are vectors for each *j* covariate for list of variables:

1. Age in years;
2. Gender (1 = female; 0 = male);
3. Marital status (1 = yes; 0 = otherwise);
4. Level of education (1 = incomplete secondary, 2 = secondary level completed with or without vocational training, and 3 = higher education);
5. Settlement of residence (1 = village, 2 = urban/small town, and 3 = city);
6. Working status (1 = employed; 0 = otherwise); and
7. Had an episode of acute illness during the last 12 months (1 = yes; 0 = otherwise).

$\beta_{1j}$ are vectors of unknown parameters and $\varepsilon_{1i}$ is error term.

(2) $$y_{2i}^{*}=\overset{7}{\underset{k=1}{\sum }}x_{2ik}^{\prime }\beta_{2k}+\gamma y_{1i}+\varepsilon_{2i}$$

where $y_{2,i}$ is a vector of perceived health status (SAH: 5 = excellent perceived health, …, 1 = very bad perceived health) and $x_{2ik}^{\prime }$ are vectors for each *k* covariate for a list of variables:

1. Duration in years of suffering with chronic disease;
2. Duration in years of disability;
3. Age in years;
4. Gender (1 = female; 0 = male);
5. Marital status (1 = yes; 0 = no);
6. Socioeconomic position (SEP; 1 = above median income; 0 = otherwise); and
7. Overall life satisfaction (1 = satisfied; 0 = otherwise), which is a control variable for possible systematic bias in the self-reported responses.

$\beta_{2k}$ and γ are vectors of unknown parameters. γ represents the effect that $y_{1i}^{*}$ has on $y_{2i}^{*}$ and $\varepsilon_{2i}$ is the error term.

(3) $$\mathrm{Cov}\;[\varepsilon_{1i},\varepsilon_{2i}|x_{1ij},x_{2ik}]=\rho$$

Two error terms, namely $\varepsilon_{1i}$ and $\varepsilon_{2i}$, are assumed to be jointly normal with correlation coefficient ρ.

Clinical morbidity is denoted by $y_{1i}$ and takes on a unitary value if diagnosed with any or a combination of the chronic diseases. SAH is an ordered discrete variable, $y_{2i}$, which takes on values from 1 to 5 (5 = excellent perceived health, 4 = good, 3 = average, 2 = bad, and 1 = very bad perceived health). Let $y_{1i}^{*}$ be the latent variable of clinical morbidity and $y_{2i}^{*}$ be the latent variable of SAH, which depend on the exogenous variables $x_{1i}$ and $x_{2i}$, respectively. Endogeneity is considered in Equation (2) which employs parameter γ. Testing the recursivity of the model is done by testing γ = 0, whereas testing the endogeneity of $y_{1i}$ in Equation (2) requires testing the hypothesis that ρ = 0 using a likelihood ratio.

The probability of taking certain discrete values depends on the cut-off points estimated. For clinical morbidity, there is only one cut-off point, $c_{1,1}$, whereas for SAH, there are four cut-off points: $c_{2,1}$, $c_{2,2}$, $c_{2,3}$, and $c_{2,4}$.

(4) $$y_{1i}=\{\begin{array}{c} 0\;if\;y_{1i}^{*}\leq c_{1,1}\\ 1\;otherwise \end{array}$$

(5) $$y_{2i}=\{\begin{array}{c} 1\;if\;y_{2i}^{*}\leq c_{2,1}\\ 2\;if\;c_{2,1}<y_{2i}^{*}\leq c_{2,2}\\ 3\;if\;c_{2,2}<y_{2i}^{*}\leq c_{2,3}\\ 4\;if\;c_{2,3}<y_{2i}^{*}\leq c_{2,4}\\ 5\;if\;c_{2,4}<y_{2i}^{*} \end{array}$$

We employed instruments to confirm the recursivity of the model and to correct for its endogeneity. Our instruments are ‘settlement of residence’ and ‘working status’. These instruments were independent of unmeasured confounders but likely induce substantial variation in the endogenous covariate, i.e., in the presence of clinical morbidity. We chose such variables that have a higher probability of an effective interaction with the health system and these variables are settlement of residence and working status but are unrelated to SAH (exclusion restriction). Such an approach identified the model parameters in a better way and thus established the robustness of the model results [28].

The model was estimated by the full information maximum likelihood function (an asymptotically efficient estimator for simultaneous models with normally distributed errors), all estimated parameters being structural.

We also fully derived the conditional probabilities and partial effects on differences in the conditional probabilities within the recursive semi-ordered probit model (Appendix B).

The mean variance inflation factor (VIF) of 2.2 suggested the stability of the coefficients and the tolerance (1/VIF) values of not lower than 0.5 for none of the predictor variables implied a substantial large contribution of each of the predictor variables used in the models.

## 3. Results

Testing the exogeneity of the clinical morbidity in the recursive semi-ordered probit model concerned testing significance of Equation (3). ρ, the ‘atanhrho’ statistic, measured Fisher’s Z transformation of the correlation between error terms from both models [29] and was strongly significant (+0.23, *p*-value = 0.000). Such strong evidence of the unobserved heterogeneity conjointly affecting the morbidity (diagnosed chronic disease) and perceived health established the endogeneity effect of clinical morbidity (as a predictor) on perceived health. Furthermore, a strong and significant likelihood ratio (LR) test (91.77, *p*-value = 0.000) established a parsimoniousness of the models used.

Table 3 (column 1) presents that the probability of having clinical morbidity was influenced by age, gender, education, marital status, settlement of residence, working status, and episode of acute illness. The effect of age was found to be significant, in tandem with the expected effects from other variables (gender, economic position, and so on).

The second part of Table 3 provides the result for SAH with the two instruments (settlement of residence and working status) used in the first step. We examined the endogeneity effect of clinical morbidity (as a predictor) on perceived health. Our variable of interest, i.e., clinical morbidity, was found to have a significant influence on the perceived health status. The recursive model displayed a much stronger negative and significant impact on perceived health (coefficient value, −1.24) after treatment of the endogeneity effect compared to the naive model (coefficient value, −0.82) implying an estimated coefficient biased with endogeneity. Most strikingly, a positive sign of our interaction variable, as captured in the recursive model (could not be captured in the naive model because being diagnosed with any one of the chronic diseases was more significant than the duration of suffering, thus the number of the levels of the interaction effect was too big compared to the diagnosed levels with any one of the chronic diseases (i.e., 0 or 1)), indicated that the duration of suffering also had a significant influence on the perceived health status.

A strong year effect on perceived health status, ceteris paribus, was evident consistently from 2004 and onwards compared to 2001, suggesting an overall influence of country environment (possibly the living conditions) on perceived health status. The year effect on SAH exhibited an increasing trend with the reference year as 2001. The minimised AIC and BIC scores favoured the recursive model (Table 3).

Estimating the recursive semi-ordered probit model allowed us to compute conditional probabilities and the differences in conditional probabilities at each given level of perceived health (Table 4). Although statistically strongly significant, conditional probabilities (condition of having or not having clinical morbidity) were almost similar in both situations for very bad perceived health status, while the differences between the conditional probabilities were substantial (even more than 20%) for the higher levels of perceived health status. Furthermore, the impact of clinical morbidity on the higher levels of perceived health status were continuously increasing from 2014 compared to the preceding year. The limited number of respondents with excellent perceived health status in our data did not allow the model to capture any effect of clinical morbidity on the probabilities of excellent perceived health status across the years. However, interestingly, the condition of having clinical morbidity did not affect the probability of good perceived health, while not having clinical morbidity increased the probability of good perceived health.

**Figure 1:**
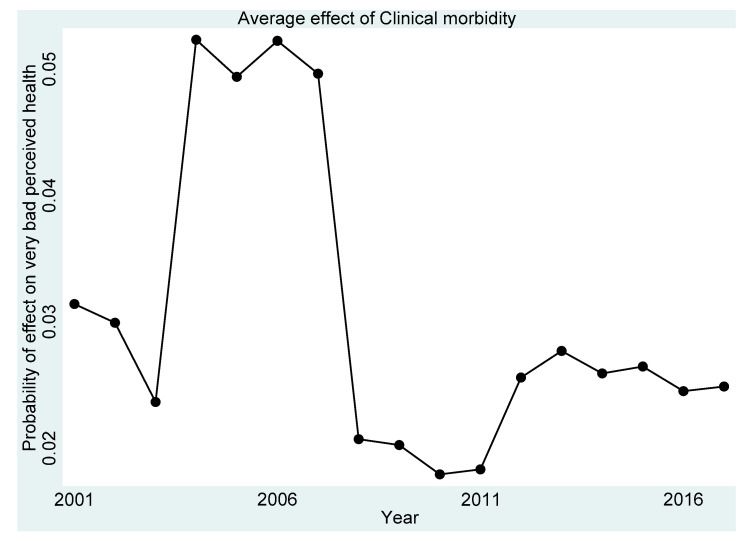
Probability of effects on “very bad” perceived health from the average effect of clinical morbidity.

**Figure 2:**
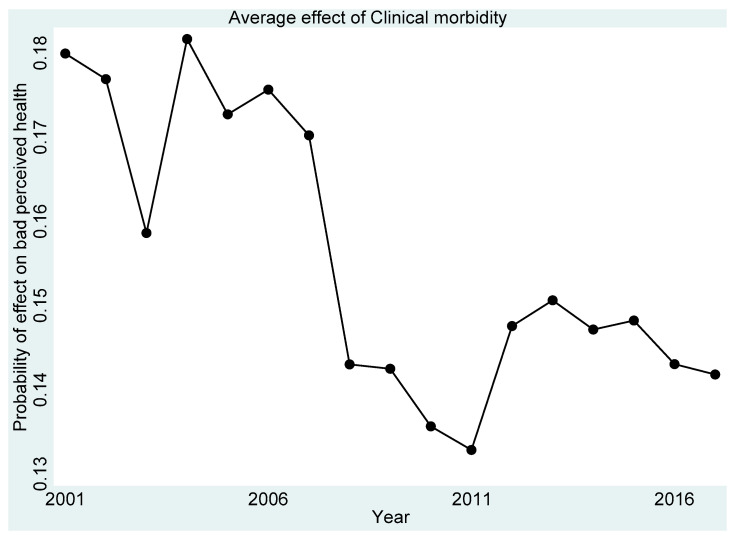
Probability of effects on “bad” perceived health from the average effect of clinical morbidity.

**Figure 3:**
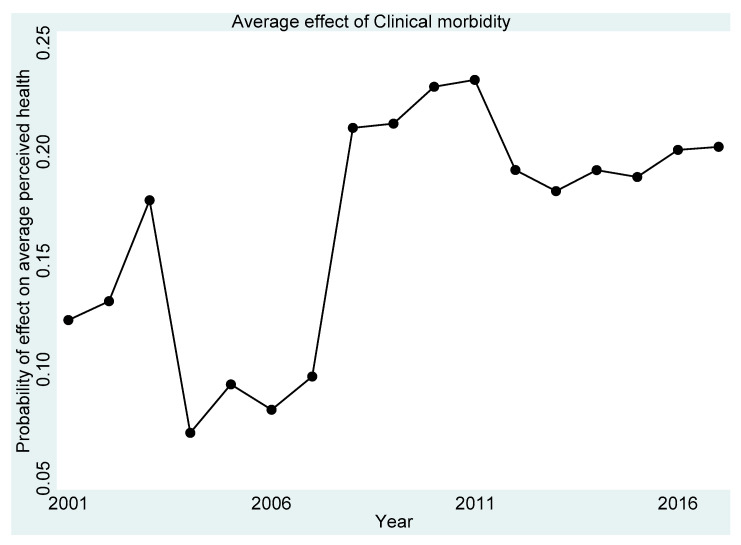
Probability of effects on “average” perceived health from the average effect of clinical morbidity.

**Figure 4:**
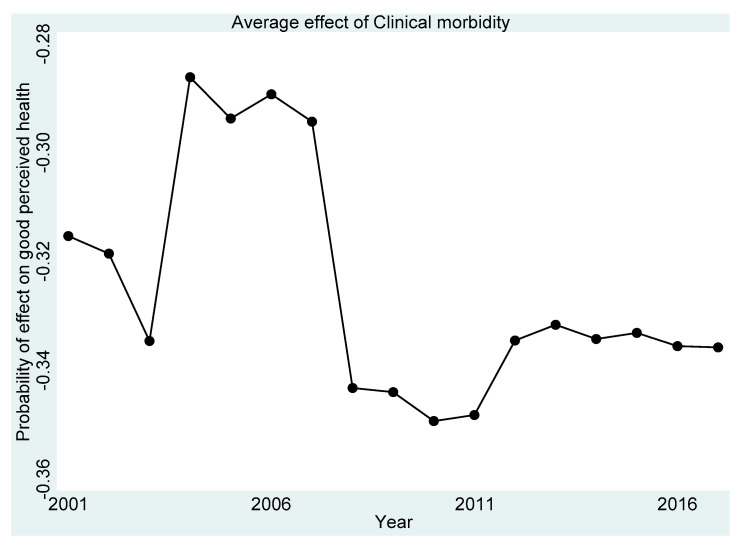
Probability of effects on “good” perceived health from the average effect of clinical morbidity.

**Figure 5:**
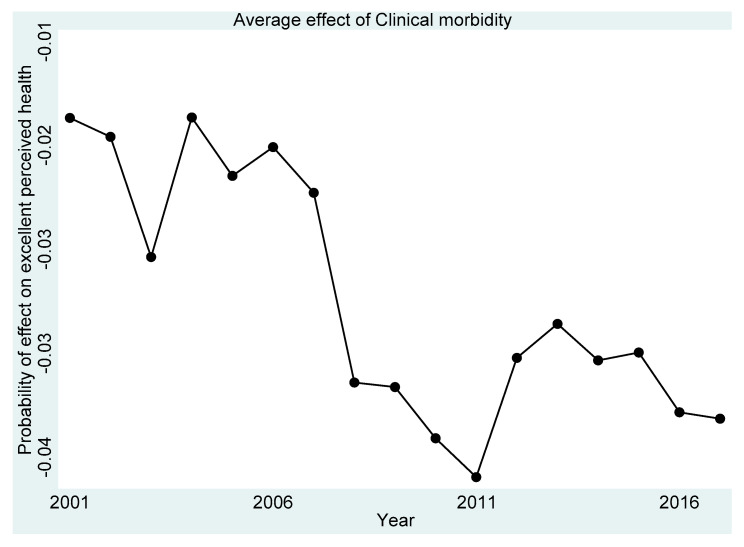
Probability of effects on “excellent” perceived health from the average effect of clinical morbidity.

Figure 1, Figure 2, Figure 3, Figure 4 and Figure 5 present the year-on-year probability of the average effect of clinical morbidity on the different levels of perceived health status when the reference year was 2001. Overall, the effect of clinical morbidity on the different levels of perceived health status was not consistent across the years. With clinical morbidity, the probability of higher levels (above average) of perceived health decreased. In general, the probability of the average effect of clinical morbidity on the probability of “average level” of perceived health status (Figure 3) compared to such effect (s) on “very bad” (Figure 1) and “bad” (Figure 2) perceived health statuses was substantially higher from 2007 and onwards.

## 4. Discussion

Reporting heterogeneity in SAH is a potentially serious problem for decision making and for achieving distributional efficiency in population health development. Thus, differential reporting by a subgroup of the population deserves an objective assessment. The concern for the reporting heterogeneity often correlated with clinical morbidity limits the unbiased use of SAH for measuring the performance of the resources allocated to the health system. Our data provided us a unique opportunity to examine the disentangled effect of a system-biased health measure, i.e., clinical morbidity (diagnosed chronic disease), on SAH. We introduced the application of a recursive model to examine reporting heterogeneity. Furthermore, we empirically validated the competitiveness of the recursive model over the classical approach.

A reasonably strong and significant correlation with a negative sign between clinical morbidity and perceived health status suggested that clinical morbidity was an endogenous regressor for SAH. The positive value of the duration of suffering interacted with the corresponding clinical morbidity, plausibly reflecting adaptation to the suffering with the chronic disease over time. This revelation confirmed a probable psychological adaptation of patients to the chronic disease they had been suffering from [8]. Such an interaction effect was possible to capture only in the recursive model without any computational stress.

Furthermore, the computation of the conditional probabilities in the framework of the recursive semi-ordered probit model established the distributional effect of clinical morbidity at different levels of perceived health. Predictions and estimations are not the same: the recursive model allowed us to estimate the conditional probabilities. Our findings confirmed that inferences from conditional probabilities were greatly superior forms of evidence for decision making.

Our study contributes further to the literature that argues SAH alone is not enough for policy conclusions, with a particular concern for the perceived health status reported as “average”, confirming the result of Au and Johnston [9]. Therefore, having a control variable tagged to the SAH question is a necessity in the survey questionnaire for effectively addressing the problem of reporting heterogeneity by subgroups of the population.

We used pooled data of 17 waves from a nationally representative sample. However, repeated observations on the same individuals were not systematic, thus it is possible that this study could not well-capture time-varying phenomena concerning the period of suffering in the construct of perceived health for individual adaptability. Our results also support the positive contributions of the instruments used, although we could not eliminate the possible recall bias in the date of diagnosis of the chronic disease. A positive and statistically strongly significant effect of the interaction variable, i.e., duration of suffering, with the clinical morbidity variable, confirmed the appropriateness of the IVs used. Moreover, the sample size used in the study was large enough to verify the stability of the IV-result across time and across different classes of SAH (levels of perceived health), which was quite reassuring.

## 5. Conclusions

To conclude, this study has introduced a new tool in the methodological approach for examining the endogeneity effect on the reporting behaviour for SAH. The methodological approach that we demonstrated can also be used for guiding distributional dimensions of the variable of interest regarding public policy performance. Controlling for endogeneity to establish a “causal” effect necessitates operationalising a simultaneous binary and ordered probit-semi ordered approach. Hence, this empirical validation of the recursive semi-ordered probit model contributes to the literature with the possibilities for replications of model use, where the aim is to estimate the distribution of the endogeneity effect on the variable of interest instead of just examining a point-estimate of the bias. Notwithstanding sample-specific results, we also highlight the policy dimension of heterogeneity in the impact of clinical morbidity on SAH. Perceived health status determines the demand for healthcare service consumption and hence the findings and approach from this study provide insights into public policy development for healthcare service production and the organisation of health systems.

## Figures and Tables

**Table 1:** Clinical morbidity and levels of perceived health status.

	Duration (Mean) of Suffering in Years	Perceived Health (%)				
Clinical Morbidity		Very Bad	Bad	Average	Good	Excellent
Heart disease (*N* = 6795)	14.96 (0.17)	7.49	37.44	51.48	3.49	0.10
Gastrointestinal disease (*N* = 6822)	15.72 (0.16)	4.63	25.49	61.24	8.46	0.18
Spinal diseases (*N* = 6495)	15.69 (0.16)	4.46	26.82	60.63	7.90	0.18
Other chronic disease (*N* = 9127)	14.42 (0.13)	5.23	27.93	57.97	8.63	0.24
Assigned disability (*N* = 6761)	9.89 (0.12)	9.50	41.15	44.56	4.61	0.18

The test of independence (ꭕ2) for all values (presence of clinical morbidity in the distribution of perceived health status) is at the *p*-value = 0.000. Figures in parentheses indicate standard error.

**Table 2:** Data used.

Survey Year	Respondents (Aged 18 Years and above)	Year-on-Year Attrition (%)
2001	4773	16.47
2002	4874	10.65
2003	4768	10.63
2004	9627	9.21
2005	9393	5.75
2006	11,460	24.15
2007	11,294	9.00
2008	6011	8.27
2009	5916	6.37
2010	8993	37.95
2011	10,074	17.22
2012	10,942	15.40
2013	10,468	10.79
2014	9097	6.41
2015	9091	6.84
2016	9136	5.81
2017	9322	5.13
Total	145,239	12.44

**Table 3:** The model results.

	Recursive Model	Naive Model
Dependent Variable = Clinical Morbidity	Coeff.	Coeff.
Age	0.018 ***	
	[(0.02)–(0.02)]	
Gender (comparison: female)		
Male	−0.202 ***	
	[(−0.22)–(−0.18)]	
Education (comparison: secondary education incomplete)		
Secondary with/without vocational training	−0.072 ***	
	[(−0.10)–(−0.05)]	
Higher education	−0.135 ***	
	[(−0.16)–(−0.11)]	
Marital status (comparison: otherwise)		
Married	−0.035 **	
	[(−0.06)–(−0.01)]	
Settlement of residence (comparison: village)		
Town	0.104 ***	
	[(0.08)–(0.13)]	
City	0.202 ***	
	[(0.18)–(0.22)]	
Working status (comparison: otherwise)		
Employed	−0.116 ***	
	[(−0.14)–(−0.10)]	
Acute illness episode (comparison: no heart attack/no stroke)		
Heart attack	1.654 ***	
	[(1.57)–(1.74)]	
Stroke	0.695 ***	
	[(0.63)–(0.76)]	
**Dependent Variable = Perceived Health Status**		
Clinical morbidity	−1.238 ***	−0.817 ***
	[(−1.32)–(−1.16)]	[(−0.85)–(−0.79)]
Duration of clinical morbidity		0.003 ***
		[(0.00)–(0.00)]
Interaction effect (duration of clinical morbidity)	0.004 ***	
	[(0.00)–(0.01)]	
Assigned with disability	−0.947 ***	−1.003 ***
	[(−0.99)–(−0.90)]	[(−1.05)–(−0.96)]
Duration of life with disability	0.009 ***	0.010 ***
	[(0.01)–(0.01)]	[(0.01)–(0.01)]
Age	−0.028 ***	−0.030 ***
	[(−0.03)–(−0.03)]	[(−0.03)–(−0.03)]
Gender (comparison: female)		
Male	0.218 ***	0.236 ***
	[(0.20)–(0.23)]	[(0.22)–(0.25)]
Marital status (comparison: otherwise)		
Married	−0.063 ***	−0.052 ***
	[(−0.08)–(−0.05)]	[(−0.07)–(−0.03)]
Economic position (comparison: below median income)		
Income above median level	0.083 ***	0.086 ***
	[(0.07)–(0.10)]	[(0.07)–(0.10)]
Satisfied with Life (comparison: otherwise)		
Satisfied with Life	0.424 ***	0.428 ***
	[(0.41)–(0.44)]	[(0.41)–(0.44)]
Year (comparison: 2001)		
2002	−0.042	−0.043
	[(−0.09)–(0.00)]	[(−0.09)–(0.01)]
2003	0.066 **	0.066 **
	[(0.02)–(0.11)]	[(0.02)–(0.12)]
2004	0.033	0.035
	[(−0.01)–(0.07)]	[(−0.01)–(0.08)]
2005	0.089 ***	0.092 ***
	[(0.05)–(0.13)]	[(0.05)–(0.14)]
2006	0.058 **	0.060 **
	[(0.02)–(0.10)]	[(0.02)–(0.10)]
2007	0.092 ***	0.095 ***
	[(0.05)–(0.13)]	[(0.05)–(0.14)]
2008	0.130 ***	0.132 ***
	[(0.09)–(0.17)]	[(0.09)–(0.18)]
2009	0.138 ***	0.141 ***
	[(0.09)–(0.18)]	[(0.09)–(0.19)]
2010	0.175 ***	0.178 ***
	[(0.13)–(0.22)]	[(0.14)–(0.22)]
2011	0.206 ***	0.210 ***
	[(0.17)–(0.25)]	[(0.17)–(0.25)]
2012	0.132 ***	0.136 ***
	[(0.09)–(0.17)]	[(0.09)–(0.18)]
2013	0.119 ***	0.123 ***
	[(0.08)–(0.16)]	[(0.08)–(0.16)]
2014	0.161 ***	0.166 ***
	[(0.12)–(0.20)]	[(0.12)–(0.21)]
2015	0.166 ***	0.171 ***
	[(0.12)–(0.21)]	[(0.13)–(0.21)]
2016	0.223 ***	0.229 ***
	[(0.18)–(0.26)]	[(0.19)–(0.27)]
2017	0.222 ***	0.229 ***
	[(0.18)–(0.26)]	[(0.19)–(0.27)]
*N*	145,239	145,239
AIC	−13.943	−12.674
BIC	−13.941	−12.623
Log-likelihood	−170,215.08	−120,338.33

Legends: **, *p*-value ≤ 0.01; and ***, *p*-value ≤ 0.001. Figures in parentheses indicate confidence interval at 95%.

**Table 4:** Conditional probabilities and difference in conditional probabilities for different levels of perceived health.

Probability	2001	2002	2003	2004	2005	2006	2007	2008	2009	2010	2011	2012	2013	2014	2015	2016	2017
PrCond(clinical morbidity = 1)	0.016 (0.001)	0.017 (0.001)	0.015 (0.001)	0.016 (0.001)	0.014 (0.001)	0.015 (0.001)	0.014 (0.001)	0.014 (0.001)	0.013 (0.001)	0.013 (0.001)	0.012 (0.001)	0.013 (0.001)	0.014 (0.001)	0.013 (0.001)	0.013 (0.001)	0.012 (0.001)	0.012 (0.001)
PrCond(clinical morbidity = 0)	0.015 (0.001)	0.016 (0.001)	0.014 (0.001)	0.014 (0.001)	0.013 (0.001)	0.014 (0.001)	0.013 (0.001)	0.012 (0.001)	0.012 (0.001)	0.012 (0.001)	0.011 (0.001)	0.012 (0.001)	0.013 (0.001)	0.012 (0.001)	0.012 (0.001)	0.011 (0.001)	0.011 (0.001)
Difference at perceived health = very bad	0.001	0.001	0.001	0.002	0.001	0.001	0.001	0.002	0.001	0.001	0.001	0.001	0.001	0.001	0.001	0.001	0.001
Prcond(clinical morbidity = 1)	0.108 (0.003)	0.112 (0.002)	0.102 (0.002)	0.105 (0.002)	0.100 (0.002)	0.103 (0.002)	0.100 (0.002)	0.097 (0.002)	0.096 (0.002)	0.093 (0.002)	0.090 (0.002)	0.096 (0.002)	0.098 (0.002)	0.094 (0.002)	0.094 (0.002)	0.089 (0.002)	0.089 (0.002)
Prcond(clinical morbidity = 0)	0.076 (0.002)	0.079 (0.002)	0.071 (0.002)	0.073 (0.001)	0.069 (0.001)	0.071 (0.001)	0.069 (0.001)	0.066 (0.001)	0.066 (0.001)	0.063 (0.001)	0.061 (0.001)	0.066 (0.001)	0.067 (0.001)	0.064 (0.001)	0.064 (0.001)	0.060 (0.001)	0.060 (0.001)
Difference at perceived health = bad	0.032	0.033	0.031	0.032	0.031	0.032	0.031	0.031	0.030	0.030	0.029	0.030	0.031	0.030	0.030	0.029	0.029
PrCond(clinical morbidity = 1)	0.875 (0.004)	0.870 (0.003)	0.883 (0.003)	0.879 (0.002)	0.885 (0.002)	0.882 (0.002)	0.886 (0.002)	0.890 (0.003)	0.891 (0.003)	0.894 (0.002)	0.898 (0.002)	0.890 (0.002)	0.889 (0.002)	0.893 (0.002)	0.894 (0.002)	0.899 (0.002)	0.899 (0.002)
PrCond(clinical morbidity = 0)	0.634 (0.006)	0.638 (0.005)	0.627 (0.005)	0.630 (0.005)	0.624 (0.005)	0.628 (0.005)	0.624 (0.005)	0.619 (0.005)	0.618 (0.005)	0.614 (0.005)	0.610 (0.005)	0.619 (0.005)	0.621 (0.005)	0.615 (0.005)	0.615 (0.005)	0.607 (0.006)	0.607 (0.006)
Difference at perceived health = average	0.241	0.232	0.256	0.249	0.261	0.254	0.262	0.271	0.273	0.28	0.288	0.271	0.268	0.278	0.279	0.292	0.292
Prcond(clinical morbidity = 1)	0.000	0.000	0.000	0.000	0.000	0.000	0.000	0.000	0.000	0.000	0.000	0.000	0.000	0.000	0.000	0.000	0.000
Prcond(clinical morbidity = 0)	0.275 (0.008)	0.266 (0.006)	0.289 (0.006)	0.282 (0.005)	0.294 (0.006)	0.287 (0.005)	0.294 (0.005)	0.302 (0.006)	0.304 (0.006)	0.312 (0.006)	0.318 (0.006)	0.303 (0.006)	0.300 (0.006)	0.309 (0.006)	0.310 (0.006)	0.322 (0.006)	0.322 (0.006)
Difference at perceived health = good	−0.275	−0.266	−0.289	−0.282	−0.294	−0.287	−0.294	−0.302	−0.304	−0.312	−0.318	−0.303	−0.300	−0.309	−0.310	−0.322	−0.322

Figures in parentheses indicate standard errors. All values are significant at a *p*-value of ≤0.001.

## Data Availability

Data used in this study comes from http://www.cpc.unc.edu/rlms, accessed on 21 February 2021.

## Appendix A

Naive model-ordered probit

The dependent variable, perceived health, is ordered in nature with the assumption of an underlying latent (true) health status of an individual, i. Thus, the ordered nature of the dependent variable directs us to use an ordered probit (OP) model. The observed scale scores on y are assumed to be discretised measurements on an otherwise continuous but latent response variable $y^{*}$.

(A1) $$y_{i}^{*}=\alpha +x_{i}\beta +u_{i}$$

$y_{i}^{*}$ = the latent index of reported health. The response variable in the model is unobserved; $y^{*}$ is connected to y through a series of cut-points.

(A2) $$y=1\;\equiv y^{*}\;\leq \;\alpha_{1}$$

(A3) $$y=2\;\equiv \;\alpha_{1}<y^{*}\;\leq \;\alpha_{2}$$

(A4) $$y=3\;\equiv \;\alpha_{2}<y^{*}\;\leq \;\alpha_{3}$$

(A5) $$y=4\;\equiv \;\alpha_{3}<y^{*}\;\leq \;\alpha_{4}$$

(A6) $$y=5\;\equiv \;y^{*}>\alpha_{4}$$

The cut point $\alpha_{j}$ partitions the latent variable for j categories of y. Hence, for a j-category response variable, j − 1 cut-points fully partition $y^{*}$. Thus, the latent index measures an individual’s scale of health. Once $y_{i}^{*}$ crosses a certain value (threshold), perceived health is reported as ‘very bad’, ‘bad’, ‘average’, ‘good’, and ‘excellent’.

(A7) $$y_{i}=j\;\Leftrightarrow k_{j-1}\leq y_{i\;}^{*}\leq \;k_{j},\;\;\;j=1,\;\ldots \;,\;5.$$

(A8) $$E\left[u_{i}\right]=0$$

(A9) $$Var\left[u_{i}\right]=0$$

Here, $k_{j}$ are the unknown threshold parameters and βs are the unknown coefficients. Thus, the threshold divides the real line ($y^{*}$) into j categories.

Observable and unobservable factors influence the latent variable ($y_{i}^{*}$ is a function of observed and unobserved variables). The OP model follows that the coefficient vector β is the same for all categories of j, implying that the independent variable can shift the cumulative distribution either to the left or right, but the slope remains constant: the probability curve changes, leaving the slope unchanged.

Table A1 of Appendix A reports the marginal effect of clinical morbidity evaluated at each level of perceived health. The negative effect of clinical morbidity on the probability of “good” perceived health was strongest across all the years.

**Table A1:** Marginal effects of clinical morbidity on perceived health of the naive model.

Marginal Effect of Clinical Morbidity	2001	2002	2003	2004	2005	2006	2007	2008	2009	2010	2011	2012	2013	2014	2015	2016	2017
Pr(perceived health status ≤ very bad), perceived health status = very bad	0.015 (0.000)	0.016 (0.000)	0.014 (0.000)	0.014 (0.000)	0.013 (0.000)	0.014 (0.000)	0.013 (0.000)	0.013 (0.000)	0.012 (0.000)	0.012 (0.000)	0.011 (0.000)	0.012 (0.000)	0.013 (0.000)	0.012 (0.000)	0.012 (0.000)	0.011 (0.000)	0.011 (0.000)
Pr(very bad < perceived health status ≤ bad), perceived health status = bad	0.067 (0.002)	0.070 (0.002)	0.063 (0.002)	0.065 (0.001)	0.061 (0.001)	0.063 (0.001)	0.061 (0.001)	0.059 (0.002)	0.058 (0.002)	0.056 (0.001)	0.054 (0.001)	0.058 (0.001)	0.059 (0.001)	0.057 (0.001)	0.056 (0.001)	0.053 (0.001)	0.053 (0.001)
Pr(bad < perceived health status ≤ average), perceived health status = average	0.178 (0.004)	0.172 (0.004)	0.185 (0.004)	0.182 (0.004)	0.188 (0.004)	0.185 (0.004)	0.188 (0.004)	0.192 (0.004)	0.193 (0.004)	0.196 (0.004)	0.199 (0.004)	0.192 (0.004)	0.192 (0.004)	0.195 (0.004)	0.196 (0.004)	0.200 (0.004)	0.200 (0.004)
Pr(average < perceived health status ≤ good), perceived health status = good	−0.227 (0.004)	−0.227 (0.004)	−0.225 (0.004)	−0.226 (0.004)	−0.224 (0.004)	−0.225 (0.004)	−0.223 (0.004)	−0.222 (0.004)	−0.221 (0.004)	−0.219 (0.004)	−0.216 (0.004)	−0.221 (0.004)	−0.222 (0.004)	−0.219 (0.004)	−0.219 (0.004)	−0.215 (0.004)	−0.215 (0.004)
Pr(good < perceived health status ≤ excellent), perceived health status = excellent	−0.033 (0.001)	−0.030 (0.001)	−0.037 (0.001)	−0.035 (0.001)	−0.039 (0.001)	−0.037 (0.001)	−0.039 (0.001)	−0.042 (0.001)	−0.042 (0.001)	−0.045 (0.001)	−0.048 (0.001)	−0.042 (0.001)	−0.041 (0.001)	−0.044 (0.001)	−0.045 (0.001)	−0.049 (0.001)	−0.049 (0.001)

Figures in parentheses indicate standard errors. All values are significant at a *p*-value of ≤0.001.

## Appendix B

Computation of partial effects on conditional probability:

(A10) $$P_{r}(y_{2i}=k\;|y_{1i}=j)=\frac{P_{r}\left(y_{1i}=j,\;y_{2i}=k\right)}{P_{r}(y_{1i}=j},$$

where the marginal univariate probability is

(A11) $$P_{r\;}\left(y_{1i}=j\right)=\Phi \left(A_{Uj}\right)-\Phi \left(A_{Lj}\right).$$

In our model specification, $y_{1i}$ takes on two values (0,1). Thus, we compute the difference in conditional probabilities, i.e.,

(A12) $$Dif_{ki}=P_{r}(y_{2i}=k\;|y_{1i}=1)-P_{r}(y_{2i}=k\;|y_{1i}=0)$$

to estimate the effect of $y_{1i}$ on $y_{2i}$.

Computing differences in conditional probabilities allows for the assessment of whether the differences are significant and allows the calculations concerning the partial effect on the differences. Therefore,

(A13) $$Dif_{ki}=\frac{P_{r\;}\left(y_{1i}=1,y_{2i}=k\right)\;}{P_{r\;}\left(y_{1i}=1\right)}-\frac{P_{r\;}\left(y_{1i}=0,y_{2i}=k\right)\;}{P_{r\;}\left(y_{1i}=0\right)}$$

The average difference is obtained by averaging the individual differences over all observations.

Computation of the partial effect, when ρ=0,

(A14) $$\begin{array}{c} \frac{\delta Dif_{ki}}{\delta x_{1}}=\;\left(\frac{\frac{\delta P_{r\;}\left(y_{1i}=1,y_{2i}=k\right)}{\delta x_{1}}\;}{P_{r\;}\left(y_{1i}=1\right)}-\;P_{r}(y_{2i}=k\;|y_{1i}=1)\frac{\Phi \left(A_{U1}\right)-\;\Phi \left(A_{L1}\right))}{P_{r\;}\left(y_{1i}=1\right)}\left(-\beta_{1}\right)\;\;\right)-\\ \left(\frac{\frac{\delta P_{r\;}\left(y_{1i}=0,y_{2i}=k\right)}{\delta x_{1}}\;}{P_{r\;}\left(y_{1i}=0\right)}+\;P_{r}(y_{2i}=k\;|y_{1i}=0)\frac{\Phi \left(A_{U0}\right)-\;\Phi \left(A_{L0}\right))}{P_{r\;}\left(y_{1i}=0\right)}\left(-\beta_{1}\right)\right)\;\mathrm{and}\; \end{array}$$

(A15) $$\frac{\delta Dif_{ki}}{\delta x_{2}}=\left(\frac{\frac{\delta P_{r\;}\left(y_{1i}=1,y_{2i}=k\right)}{\delta x_{2}}\;}{P_{r\;}\left(y_{1i}=1\right)}-\frac{\frac{\delta P_{r\;}\left(y_{1i}=0,y_{2i}=k\right)}{\delta x_{2}}\;}{P_{r\;}\left(y_{1i}=0\right)}\right)$$

Thus, the marginal effect of $y_{1i}$ on $y_{2i}$ is

(A16) $$\begin{array}{c} DΙfk_{i}=\frac{P_{r\;}\left(y_{1i}=1,y_{2i}=k\right)}{P_{r\;}\left(y_{1i}=1\right)}-\frac{P_{r\;}\left(y_{1i}=0,y_{2i}=k\right)}{P_{r\;}\left(y_{1i}=0\right)}\;=\frac{\;P_{r\;}\left(y_{1i}=1\right)x\;P_{r\;}\left(y_{2i}=k|y_{1i}=1\right)}{P_{r\;}\left(y_{1i}=1\right)}-\;\\ \frac{P_{r\;}\left(y_{1i}=0\right)x\;P_{r\;}\left(y_{2i}=k|y_{1i}=0\right)}{P_{r\;}\left(y_{1i}=0\right)} \end{array}$$

(A17) $$=P_{r\;}\left(y_{2i}=k|y_{1i}=1\right)-P_{r\;}\left(y_{2i}=k|y_{1i}=0\right).$$
